# Virtual care delivery in Saskatchewan: Multi-stakeholder perspectives on implementation, appropriateness, and evaluation

**DOI:** 10.1177/08404704251348858

**Published:** 2025-06-18

**Authors:** Sarah-Marie Durr, Abd Alras, Stacey Lovo, Hamza Dani, Laureen McIntyre, Amy Zarzeczny, Paul Babyn, Scott J. Adams, Ivar Mendez

**Affiliations:** 1 7235 University of Saskatchewan, Saskatoon, Saskatchewan, Canada.; 2 Virtual Health Hub, Saskatoon, Saskatchewan, Canada.; 3 6846 University of Regina, Regina, Saskatchewan, Canada.

## Abstract

The purpose of this study was to provide an update on patients’, clinicians’, and health administrators’ experiences and perspectives on opportunities, barriers, and priorities for virtual care to inform health policy and planning as virtual care programs continue to mature and develop. Three surveys were developed and distributed in Saskatchewan, Canada. Quantitative data were analyzed using descriptive statistics and chi-squared tests, and free-text responses were analyzed using thematic analysis. Chronic disease management and mental health disorders were identified as highly suitable for virtual care. Health administrators underscored cost savings and improved patient access as key advantages, though they lacked consistent frameworks to assess virtual care effectiveness. Key barriers included digital literacy, technology constraints, and compensation models not aligned with virtual service provision. Participants called for greater infrastructure investment, technical support, and integrated electronic platforms. These insights may inform policy and practice to strengthen virtual health delivery and support health equity.

## Introduction

Virtual care has been defined as any interaction between patients and healthcare professionals occurring remotely that uses any form of communication or information technology.^1^ The use of virtual care skyrocketed during the coronavirus disease 2019 (COVID-19) pandemic as it became a ubiquitous form of healthcare delivery to reduce disease transmission.^2^ During the first 5 months of the COVID-19 pandemic (March-July 2020), 71.1% of all primary care visits in Ontario were virtual, as compared to 1.2% of visits during the same time period in 2019,^3^ and subsequently levelling to approximately 30%.^4^

Now in a post-pandemic world, virtual care programs can be tailored to increase access to care, quality of care, and efficiency for health systems. Although the benefits of virtual care, including increased access, cost savings, and efficiency^5-14^ are widely reported, existing research is bounded by geographic, population, and temporal contexts, and there is a paucity of literature on multi-stakeholder perspectives on the current use of virtual care.^15,16^ As virtual care programs evolve beyond those rapidly developed during the COVID-19 pandemic, there is a need for new insights that reflect current challenges and priorities. Saskatchewan, a province in Canada, is uniquely suited for virtual care, as it has a sparse population density of 2.0 people per square kilometre^17^ and nearly one-third of Saskatchewan residents live in rural communities.^17,18^ Given this demographic and geographic context, understanding how virtual care is currently experienced and perceived within the province can offer valuable insights. The objective of this research was to provide an update on patients’, clinicians’, and health system administrators’ experiences and perspectives on opportunities, barriers, and priorities for virtual care to inform health policy and planning as virtual care programs continue to mature and develop.

## Methods

### Survey development

Three separate surveys were developed for patients, clinicians, and health system administrators. We formed working groups centred around appropriateness, equity, evaluation, and implementation of virtual care. Working groups were comprised of clinicians (from medicine, nursing, physical therapy, speech-language pathology, and psychology), health leaders, a legal and policy expert, and health researchers. Each working group developed questions for each survey; among the questions included in the surveys, approximately 75% were multiple-choice while the remainder were open-ended questions to allow for more nuanced insights. Questions were piloted with a small group of participants prior to survey distribution. Complete survey questions can be obtained by contacting the corresponding authors.

### Survey sample and distribution

The patient survey was electronically distributed by the Canadian Hub for Applied and Social Research (CHASR) to a panel of Saskatchewan residents previously identified through random probability sampling. The clinician and health system administrator surveys were electronically distributed via professional and provincial health organizations and an invitation to participate in the surveys was also included on Facebook. Survey responses were collected from April 23, 2024 to June 19, 2024.

### Data analysis

Descriptive statistics were determined; categorical variables were represented as counts and proportions and continuous variables were represented as medians and interquartile ranges. Responses across demographic groups were compared using chi-squared tests. *P*-values <0.05 were considered statistically significant. Quantitative analysis was performed using SPSS version 28.0.1.0 (IBM).

Free-text responses regarding the use, benefits and limitations of virtual care were analyzed using thematic analysis.^19,20^ Free text was uploaded to NVivo 12 (QSR International). Free-text responses were reviewed and coded, and the resulting codes were analyzed to develop themes. Initial themes were jointly reviewed by two researchers to ensure compatibility with the codes and dataset. Final themes were refined and confirmed through consensus.

## Results

### Participant demographics

A total of 506 patients, 50 clinicians, and 9 health system administrators responded to the respective surveys. Demographic data for participants are presented in Table 1.

**Table 1.** Participant demographic characteristics.

		No. (%)
Patient characteristic		
Age (years)	20-29	5 (1)
	30-39	13 (3)
	40-49	62 (12)
	50-59	81 (16)
	60-69	185 (37)
	70-79	132 (26)
	80-89	28 (6)
Gender	Man	172 (34)
	Woman	326 (65)
	Agender/Nonbinary	3 (1)
	Questioning	2 (0)
	Transgender/Two-spirit	2 (0)
	Bigender/Multigender/Gender-fluid/Gender queer	0 (0)
	I prefer not to answer	1 (0)
	Other	4 (1)
Race	Caucasian	448 (88)
	First Nations/Métis	13 (3)
	Middle Eastern/West Asian	4 (1)
	South Asian	1 (0)
	East Asian	1 (0)
	South American	1 (0)
	Jewish	1 (0)
	Black	1 (0)
	I prefer not to answer	39 (8)
Type of residence	Urban	336 (68)
	Rural	161 (32)
	Remote	4 (1)
Geographic region	Far North	3 (1)
	Northwest	26 (5)
	Northeast	28 (6)
	Central	317 (64)
	Southeast	75 (15)
	Southwest	51 (10)
Clinician characteristics		
Professional role	Family physician/General practitioner	7 (14)
	Specialist physician	7 (14)
	RN or LPN	3 (6)
	Nurse practitioner	1 (2)
	Physical therapist	29 (58)
	Pharmacists/Occupational therapist/Speech-language pathologist/Psychologist/Dentist or dental specialist/Social worker	0 (0)
Location of primary practice	Urban	34 (68)
	Rural	13 (26)
	Remote	3 (6)
	Regional	0 (0)
Geographic region of primary practice	Far North	0 (0)
	Northwest	6 (12)
	Northeast	3 (6)
	Central	32 (64)
	Southeast	7 (14)
	Southwest	2 (4)
Health administrator demographics		
Area of leadership	Health authority management/Physician leadership	4 (44)
	Clinic administration	3 (33)
	Quality improvement/Support services	2 (22)
Primary location of work	Urban	5 (55)
	Rural	3 (33)
	Remote	1 (11)
	Regional	0 (0)
Scope of responsibility	Single clinic or hospital	1 (11)
	Network	1 (11)
	Area	3 (33)
	Provincial	3 (33)
	Supervisor/Manager/Director	6 (66)
	Vice-president or CEO	1 (11)
	Area department/Division lead/Area chief of staff, deputy chief medical officer, or chief medical officer	2 (22)
	Clerical/Executive director/Provincial department head	0 (0)
	Other	1 (11)

### Patient survey quantitative results

In the last 5 years, 283 (57%) patients reported receiving virtual care. Of patients who used virtual care, 212 (75%) had seen their family physician/general practitioner through virtual care and 87 (31%) had seen a specialist physician. Nurse practitioners (n = 39 [14%]), registered nurses or licenced practical nurses (n = 28 [10%]), and pharmacists (n = 27 [10%]) were also seen virtually by patients. Patients also reported seeing physical therapists (n = 16 [6%]), psychologists (n = 18 [6%]), dentists or dental specialists (n = 10 [4%]), and social workers (n = 11 [4%]) virtually.

Most patients (n = 284 [64%]) agreed that virtual care is an acceptable way to receive healthcare services, while 35 (8%) disagreed, and 126 (28%) were unsure. Patients who agreed that virtual care is an acceptable delivery modality tended to be younger (*P* < 0.01); no statistically significant differences were found with regards to gender, urban or rural residence, or ethnicity (Table 2). Of the surveyed patients, 128 (46%) were very satisfied with their previous experience of virtual care, 95 (34%) were mostly satisfied, 39 (14%) were somewhat satisfied, and 15 (5%) were not at all satisfied; no statistically significant differences were found across age groups.

**Table 2.** Patient perspectives on virtual care by demographic characteristics.

Characteristactic	Patients agree virtual care is an acceptable delivery modality, No. (%)	OR (95% CI)	Patients felt satisfied with their previous virtual care experience, No. (%)	OR (95% CI)
Age (years)				
80-89	9 (2)	Ref.	9 (3)	Ref.
70-79	73 (16)	2.77 (1.12-6.85)	68 (25)	3.78 (0.60-23.65)
60-69	102 (23)	2.70 (1.11-6.53)	93 (34)	5.17 (0.83-32.21)
50-59	48 (11)	4.21 (1.58-11.25)	37 (13)	2.06 (0.32-13.04)
40-49	39 (9)	3.61 (1.33-9.79)	43 (16)	9.56 (0.78-117.08)
<40	13 (3)	10.33 (1.97-59.46)	12 (4)	∞
Gender				
Male	90 (18)	Ref.	71 (26)	Ref.
Female	192 (39)	0.88 (0.60-1.30)	190 (69)	1.78 (0.61-5.19)
Diverse^a^				
Type of residence				
Urban	195 (44)	Ref.	175 (63)	Ref.
Rural	88 (20)	0.87 (0.58-1.31)	88 (31)	2.01 (0.55-7.31)
Remote^a^				
Geographic region				
Central	191 (43)	Ref.	169 (60)	Ref.
Northwest	10 (2)	0.34 (0.14-0.79)	8 (3)	0.17 (0.03-0.93)
Northeast	16 (4)	0.94 (0.39-2.28)	16 (6)	0.05 (0.01-0.25)
Southeast	50 (11)	1.12 (0.64-1.98)	43 (15)	∞
Southwest	21 (5)	0.41 (0.22-0.78)	28 (10)	0.02 (0.00-0.06)
Far North^a^				
Ethnicity				
Caucasian	257 (58)	Ref.	240 (54)	Ref.
Racialized	27 (6)	0.83 (0.44-1.57)	22 (5)	0.60 (0.13-2.81)

Ref. denotes the reference category.

^a^Cell sizes <3 have been suppressed. OR, odds ratio. CI, confidence interval.

The majority of patients (n = 340 [71%]) indicated that simplicity and ease of use was the most important factor when utilizing virtual care. Additionally, many patients emphasized the importance of being able to easily talk with the clinician (n = 310 [65%]) and clearly hear the clinician (n = 306 [64%]). Conversely, the least prioritized aspect was the ability to clearly see the clinician, with only 144 (30%) patients choosing this option.

The most frequently cited benefit of virtual care was reduced travel time compared to visiting a hospital or clinic, with 186 (47%) ranking it as the top advantage. Virtual care adequately providing for the patients’ needs was next most highly ranked benefit (n = 81 [21%]). Conversely, being assured of a private and secure connection was ranked as the least important, with 138 patients (36%) placing this last.

When asked the importance of virtual care options being widely available in Saskatchewan’s publicly funded healthcare system, 241 (55%) patients believed it is extremely important, 178 (41%) responded it was important or somewhat important; 23 (5%) responded it was not important.

### Clinician survey quantitative results

Forty-six clinicians (92%) reported that they had provided virtual care in the past 5 years. Among all clinicians, 31 (67%) managed chronic disease through virtual care and 20 (43%) completed periodic health visits virtually. Clinicians were most likely to list chronic disease management as most suitable for virtual care, followed by mental health disorders, periodic healthcare visits, and minor acute complaints, with life-threatening acute care as least suitable. In addition, clinicians indicated that young adults aged 19-40 have most effectively utilized virtual care, followed by older adults aged 41-64, seniors over 65, adolescents aged 13-18, and lastly children aged 0-12. When asked if virtual care has improved team-based practice for patients or improved access to specialized care teams, 16 (33%) clinicians believed it has very much and 8 (16%) said it has moderately, while 25 (51%) said it has minimally improved it.

The most useful additional support for virtual care that clinicians felt necessary was a virtual care platform integrated with electronic medical records (n = 31 [66%]), followed by technical support to assist patients in the set-up of the technology on their end (n = 29 [62%]) and technical support for their own practice (n = 17 [36%]). Most clinicians (n = 28 [56%]) said they have received adequate training to use virtual care, 14 (28%) felt they did not have adequate training, and 8 (16%) were unsure.

Fourteen clinicians (29%) believed current compensation models do not provide adequate compensation for virtual care as compared to traditional in-person care, 17 (35%) believe the current models do work well for some forms of virtual care, but more nuanced models are needed to appropriately compensate providers for other types of virtual care interactions, and 17 (35%) thought that the current models are set up to incentivize virtual care delivery.

When asked to rank the most influential barriers to comprehensive virtual care in Saskatchewan, the highest scored responses among clinicians were technology (n = 15 [31%]), socioeconomic factors (n = 11 [23%]), and cost (n = 7 [15%]), followed by compensation models, culture, regulatory and legal barriers, and education.

### Health system administrator survey quantitative results

Health system administrators were most likely to list cost savings and patient access to healthcare as the greatest benefits of virtual care (both n = 8 [89%]), followed by increasing access to teams and specialists (n = 6 [67%]). When evaluating the effectiveness of virtual care, health system administrators indicated they often review user feedback, but do not have tools or frameworks that they use to evaluate its effectiveness. Health system administrators believe increased quality of care as most important when considering implementing virtual care, followed by cost effectiveness, and lastly time effectiveness. Patients feeling unsure about the use of virtual care and limited technical support available were chosen as the most common barriers that exist which limit the uptake of virtual care (n = 8 [89%] and n = 7 [78%], respectively).

The majority (n = 7 [78%]) of health system administrators also believe virtual care can help with the known challenge of providing care in rural and remote locations, with 4 (44%) citing how it can allow for access to urgent care experts in a short time frame and 3 (33%) believing it can allow for rural and remote professionals to connect with teams, colleagues, and specialists. Nonetheless, 1 (11%) health system administrator believed virtual care would not address the challenges of providing care in rural and remote locations.

Most health system administrators (n = 5 [56%]) believed virtual care can allow for enhanced training for new professionals, and the same proportion believed it can allow for mentorship in rural and remote areas which may in turn enhance recruitment and retention. Additionally, 8 (89%) health system administrators believe virtual care can allow for some services to be provided remotely from locations where there are sufficient healthcare providers already available, while 1 (11%) thought virtual care does not impact workforce planning.

Health system administrators listed the most influential barriers to comprehensive virtual care as technology (e.g., poor data interoperability and insufficient information technology infrastructure), cost and socioeconomic factors (e.g., low digital literacy among some patients, inequitable access to the necessary technology, and language barriers) tied in second, followed by regulatory and legal barriers, culture, and education all tied for third, and lastly compensation models.

### Qualitative findings

A total of 169 patients, 26 clinicians, and 3 administrators responded to the open-ended questions. Analysis of these responses revealed five major themes and 17 additional subthemes in patients’, clinicians’, and administrators’ perspectives on virtual care (Table 3).

**Table 3.** Major themes and subthemes in patients’, clinicians’, and health system administrators’ opinions of virtual care.

Theme	Subthemes and key findings	Number of references	Illustrative quotations	Source
System requirements to ensure best practices	Proper infrastructure: The proper system, procedures, and equipment to track, book, and conduct virtual care	9 clinicians	We desperately need a patient portal as it is very time consuming for my secretary to book and collect patient forms. We also need a method for group patient education	Clinician
			Having a centralized, supported and integrated system would significantly improve the use of virtual care, in my opinion	Clinician
	Service awareness: Increasing public awareness about the option of virtual care and how to use it	6 patients	More awareness of the services &/or limitations of the services be advertised and steps taken to ensure the public is aware of the benefits. People should be encouraged to access it more. I don’t think most folks are aware of how beneficial this health phone service can be or how diverse it is	Patient
	Billing: Equal billing for in-person and virtual care to prevent provider bias	3 clinicians, 5 patients	I think it is very important that compensation models are equitable to in person. Although I have provided this care, I do know that at this has been at times resulted in 25%–45% less income and would discourage physicians as we have increased costs	Clinician
			We have a team of professionals that currently have the capacity to expand that delivery of those services but there are no billing codes to support us. If that was to change, we could expand access to care immediately	Clinician
			Doctors have communicated to me, and at times you can tell even when they don’t—that they are financially punished for providing virtual care	Patient
	Supportive role: Virtual care to support, not replace in-person care	3 clinicians, 18 patients	Virtual care is not suitable for all types of care however, when it is suitable, it is very, very useful	Clinician
			While virtual care might be a viable supplemental program. I believe that the public healthcare system requires additional restructuring to improve in-person care as well	Patient
Barriers to tackle	Communication barriers: Addressing communication barriers such as accent and language	4 patients	It is extremely important that providers of virtual care have a good command of Canadian English because a strong foreign accent which makes it difficult to communicate virtually	Patient
			Language issues are an issue as more immigrants arrive	Patient
	Digital literacy: Patients with limited digital literacy will struggle to access and use virtual care	11 patients	I am more easily frustrated with technology as I age	Patient
			Old people, those who may be the high users of the system, have problems using the technology or don’t want to use it	Patient
	Privacy: How to prevent and minimize a privacy breach during virtual care	1 clinician, 2 patients	I think privacy will have to be reviewed because I can see where the healthcare professional may be interacting with the client but no be aware there are other people in the room unannounced	Patient
	Technical difficulties and technology shortage: Troubleshooting issues can be exhaustive and time consuming for both patients and providers	2 clinicians, 7 patients	It is important to have smooth and sufficiently capable technology on both ends for successful virtual care	Clinician
Patient-provider interaction	Virtual care enhances patient-provider interaction	8 patients	During Covid, I used telephone appts with my provider and was happy with the timeliness	Patient
	Virtual care diminishes patient-provider interaction	4 patients	More often than not, people feel heard and taken care of by a provider in real life. Very limited where good care can be provided “virtually”	Patient
	Autonomy: Maintaining patient autonomy to choose the type of care	1 patient	Patient should be given the option to opt out of such a thing	Patient
Benefits of virtual care	Improved access—rural	6 clinicians, 27 patients	My small home community has very limited medical resources and would greatly benefit from the development of comprehensive virtual care	Clinician
			Wish our doctor would offer virtual care instead of sitting in an office for 2 hours and driving 11/2 hrs for her to tell you your potassium is low and you should eat more bananas	Patient
	Improved access—non-rural	1 clinician, 1 admin, 11 patients	Waiting in a doctors’ office full of illnesses can be stressful for the elderly and immuno-compromised. Also nice not to have to find parking and walk to office when icy and cold. I think both in office and virtual/phone appts allow flexibility for both the doctor and patient. My doctor was able to keep her appts using the phone when she herself was sick	Patient
			When having small children it is difficult to leave a home	Patient
			Exceptional tool for complex case conferencing	Patient
	Reduced wait times	2 clinicians, 12 patients	This has been an extremely helpful tool in providing care to my patient panel particularly by giving them quicker access to medical expertise	Clinician
			It would be an amazing resource for parents when their child is sick, rather than waiting at a walk-in clinic for hours	Patient
Time point in care	First visit vs. follow-up	1 clinician, 11 patients	It is excellent for follow-up to support compliance, process care and optimize care. For team-based care, it is excellent for case conferencing and team reviews	Clinician
	Chronic disease management	2 clinicians, 6 patients	Virtual care works well for situations such as prescription renewals and minor ailments	Patient
	Follow-up of results	1 patient	I only get phone calls about cancer test results	Patient

#### System requirements to ensure best practice

Nine clinicians mentioned the need for proper infrastructure, including systems, procedures, and equipment to effectively track, book, and conduct virtual care. Six patients mentioned the need to increase public awareness about virtual care options and their usage. Equal billing for in-person and virtual care, cited by both clinicians and patients, was noted as necessary to prevent provider bias. Lastly, 3 clinicians and 18 patients mentioned the role of virtual care as a supportive tool, complementing rather than replacing in-person care.

#### Barriers to virtual care

The most commonly referenced barrier to accessing virtual care was digital literacy. Five references were made to communication being a barrier to virtual care, which could be exacerbated by accents and language fluency issues between patients and providers. Technical difficulties and lack of access to technology were cited as barriers to the widespread adoption of virtual care.

#### Benefits of virtual care

The benefits of virtual health were categorized into rural-related and non-rural-related advantages. For rural areas, virtual care significantly reduced the burden on patients by saving time and money that would be spent travelling. It also enhanced access to primary and specialist care. Non-rural residents highlighted benefits such as reduced risk of spreading infectious diseases.

#### Patient-provider interactions

Some patients praised the efficient interactions they had with their providers, while others found virtual interactions to be impersonal, missing the face-to-face connection of traditional visits. Several patients emphasized the importance of establishing a relationship before transitioning to virtual care.

#### Time point in care

Several patients felt that virtual care was beneficial for medication refills and follow-up appointments, where physical examinations were not crucial. There was broad agreement among patients and providers that the successful implementation of virtual health hinges on choosing appropriate clinical presentations for virtual care.

## Discussion

### Need for virtual care

Virtual care experienced a dramatic and rapid expansion during the COVID-19 pandemic that has since stabilized but remains well above pre-pandemic levels. This research invited stakeholders to comment on their use and experience with virtual care to inform recommendations for practice and policy going forward. Findings from this study provide health leaders and policy-makers with updated, evidence-based insights into the experiences, barriers, and priorities of multiple stakeholder groups regarding virtual care in a post-pandemic context.

The majority of respondents from all three stakeholder groups (patients, clinicians, and health system administrators) believed that it is extremely important for virtual care options to be widely available in Saskatchewan’s publicly funded healthcare system. Findings from this study point towards specific areas of focus, including ensuring appropriate infrastructure for virtual care, including the proper platform, procedures, and equipment to track, book, and conduct virtual care; increasing public awareness about the availability of virtual care and how to use it; and ensuring appropriate compensation for virtual care. Clinicians stressed the importance of having a provincial platform and infrastructure for virtual care, suggesting greater investments are necessary to support the development of an integrated system allowing for supported, standardized virtual care across provincial health systems. The study also identified that a nuanced approach to virtual and in-person care delivery is required so that virtual care supports, but does not replace, in-person care.

### Barriers to implementing virtual care

Our results help contextualize the current level of utilization of virtual care. The majority of clinicians want to provide more virtual care in their practice, but cite many barriers including lack of technical expertise or tools and compensation models needing to be altered to make it financially attractive or viable. One patient explained virtual care billing should not become more lucrative than in-person care so that clinicians are not “biased toward virtual calls,” and with a limited clinician supply in most settings, a careful balance must be found between virtual and in-person care compensation models. Previous research has also suggested that models which result in differential compensation whether a patient has been previously seen by a clinician in-person may result in further inequities for patients without a family physician.^4^

### Benefits of virtual care

Both clinicians and patients agreed virtual care improves access to primary and specialized care for patients in rural and remote communities, highlighting a major opportunity for the implementation of virtual care. Virtual care would minimize barriers related to travel for these patients. One clinician explained that their “small home community has very limited medical resources and would greatly benefit from the development of comprehensive virtual care.” Rural and remote patients face inequities with accessing healthcare and especially specialists, which can lead to poorer health outcomes.^5^ Implementing virtual care initiatives on a larger scale could have substantial benefits, particularly for provinces like Saskatchewan which have low population density and relatively large number of rural communities.^17,18^

The theme of access was also identified in non-rural settings, with 56% of patients citing the ability to quickly see a healthcare provider as the most important advantage of virtual care, and 63% of patients responding they would use virtual care again because it took less time. The focus of time for patients is not surprising given the landscape of wait times in Canada. The current wait time from a referral to treatment initiation is at an all-time high with a median of 27.7 weeks.^21^ The national average for the wait time in the emergency department for an initial assessment by a physician is 5.0 hours, while the average for Saskatchewan is slightly better at 3.8 hours.^22^ Worryingly, Canadian wait times seem to be worse for low-income patients compared to high-income patients.^23^ Virtual care is seen among patients as a possible solution for long wait times to access care. Further research will be required to explore how policy changes which increase virtual care capacity impacts in-person care capacity and demand.

Findings surrounding the type of visits which are most suitable for virtual care provide preliminary evidence to help optimize clinical pathways and guide patients towards the care modality (virtual vs. in-person care) that may be most efficient and lead to the best outcomes. Additionally, findings that young adults have most effectively utilized virtual care compared to older adults, adolescents, and children underscores the need for targeted strategies—such as tailored training, user-friendly technologies, and enhanced support mechanisms—to improve adoption and satisfaction rates across different age groups.

An initiative working to address these challenges and opportunities is the Virtual Health Hub (VHH), an initiative based in Saskatchewan. The VHH is creating a province-wide command centre for virtual care delivery, building infrastructure and capacity for scalable virtual care in Saskatchewan to increase access to care.^24^ The VHH leverages technologies such as artificial intelligence, remote presence robotics, and remote diagnostic imaging to deliver services to patients in their home communities, reducing the need for travel to urban centres. In parallel, the VHH is building capacity through training programs for virtual health assistants in partnership with the Saskatchewan Indian Institute of Technologies, aiming to increase the number of Indigenous professionals involved in virtual care delivery. These efforts align with the identified needs for better technical support and workforce development identified in this study. To further guide implementation, the VHH has created a community-informed evaluation tool that assesses readiness across domains such as clinical need, technological infrastructure, and health system workflows.^25^ This structured and context-sensitive approach offers a promising model for overcoming the fragmented infrastructure and digital literacy gaps identified in this study, particularly in rural and remote communities.

### Study limitations

This study has some limitations. Because recruitment relied on social media and distribution through professional associations, the total number of individuals who received the survey invitation remains unknown, making it impossible to calculate a survey response rate. We also note that only nine health administrators responded to the survey, which may not capture the full diversity of opinions of this group. Additionally, participation was based on self-selection, introducing potential selection bias if those with stronger opinions about virtual care were more likely to respond. Finally, our findings are limited to a single Canadian province, and findings may not be generalizable to other geographic regions.

## Conclusion

In conclusion, our findings highlight that virtual care is particularly valued for its potential to ease access barriers, reduce travel time, and accommodate patient preferences. Despite these advantages, technology infrastructure, digital literacy, and compensation models remain significant hurdles for both patients and clinicians. Moving forward, standardized provincial platforms, robust technical support, and comprehensive training could better integrate virtual care into routine practice, ensuring its sustainability and fostering equitable care delivery.

## Data Availability

The datasets generated during and/or analyzed during the current study are available from the corresponding author on reasonable request.*
